# Development of Contextually-relevant Sexuality Education: Lessons from a Comprehensive Review of Adolescent Sexuality Education Across Cultures

**DOI:** 10.3390/ijerph16040621

**Published:** 2019-02-20

**Authors:** Hildie Leung, Daniel T. L. Shek, Edvina Leung, Esther Y. W. Shek

**Affiliations:** Department of Applied Social Sciences, The Hong Kong Polytechnic University, Hong Kong, China; daniel.shek@polyu.edu.hk (D.T.L.S.); edvina.leung@polyu.edu.hk (E.L.); catowlshek@yahoo.com.hk (E.Y.W.S.)

**Keywords:** sex education, adolescents, sexual health, sex and relationship education, sexual wellbeing

## Abstract

As reported by the World Health Organization in 2017, there are 2 million+ young people living with HIV worldwide. The World Health Organization also reported that a third of all new HIV infections around the world are estimated to occur among youths (aged 15–25). and teen pregnancy rates are on the rise in many places. These worrying trends suggest that existing sexuality education programs and interventions may be inadequate and/or ineffective. Although the 1994 International Conference on Population and Development’s (ICPD) Programme of Action highlighted the roles of Governments to offer sex education to young people to promote teenage reproductive health, yet inconsistency exists in the related initiatives in the global context. The present article aims to provide a comprehensive literature review of the existing sexuality programs in selected places in both English-speaking (i.e., the United States of America, the United Kingdom) and Chinese-speaking contexts (i.e., Hong Kong, Mainland China, and Taiwan). Based on the review, observations and implications for sexuality education policy and practice, as well as recommendations for future research for youths are outlined.

## 1. Introduction

Adolescence marks a developmental phase where one has relatively sound health, a time where physical sexual maturity is acquired. However, it is also during this period marked by increased autonomy, social immaturity, risk taking, and spontaneity which make them more susceptible to reproduction and sexual health risks. These risks include unplanned, or unprotected sex, which may lead to an elevated risk of sexually transmitted infections (STIs), unintended pregnancy, and unsafe abortion [1,2]. Although STIs and sexually transmitted diseases (STDs) are sometimes used interchangeably, it is important to highlight the technical differences between the two. STIs refer to bacteria, viruses, and parasites transmitted through vaginal, anal, and oral sex. It is a sexually transmitted infection that has not developed into a disease. Simply put, STDs (e.g., pelvic inflammatory disease, cervical cancer) start out as STIs. At present, four curable STIs include syphilis, gonorrhea, chlamydia, and trichomoniasis, while, hepatitis B, herpes, HIV, and human papillomavirus (HPV) are incurable. These pathogens are associated with higher incidence of STDs [3]. As reported by the World Health Organization [3], there are currently more than 2 million adolescents living with HIV worldwide. In the United States (U.S.), young people (aged 15–24 years old) account for 50% of all new STDs. Furthermore, 25% of sexually active adolescent females have an STD (e.g., chlamydia or HPV) [4]. However, in the present study, we will focus on providing a broader picture, as such the rates of particular types of sexually transmitted infections and diseases will not be outlined in detail. According to the 2017 national Youth Risk Behavior Survey [4], 39.5% of high school students in the U.S. reported ever having engaged in sexual intercourse. While the figures have decreased significantly in the past decade from 47.8% in 2007, unfortunately, those who reported using a condom during their last sexual contact decreased significantly from 61.5% in 2007 to 46.2% in 2017. Moreover, 70.6% of U.S. high school students did not use birth control pills, implants, or birth control rings before last intercourse. 

In non-Western contexts such as Hong Kong, the Department of Health reported that HIV infection cases increased from 181 in 1997 to 692 in 2016 [5]. The premarital pregnancy rate among youngsters had also increased from 2011 to 2016 [6]. Furthermore, improved health and nutrition in most developed countries have resulted in youths maturing at a younger age, accompanied by an earlier sexual debut [7]. In the UK, The National Survey of Sexual Attitudes and Lifestyle showed that roughly three-tenths of young people aged 16–24 had sexual intercourse when they were younger than 16 [8].

In fact, the resolution of the International Conference on Population and Development’s (ICPD) Programme of Action adopted in 1994 underscored the importance of governments to offer sex education to young people [9]. A critical need for young people to obtain information and skills to protect adolescent sexual and reproductive health (ASRH) was recognized [10]. Consequently, many countries have adopted sex education policies aimed at preventing adolescent unintended pregnancy, unsafe abortions, and HIV transmission. In the United Nations Global Strategy for Women’s, Children’s and Adolescents’ Health 2016, strategies to guide and catalyze global collaboration on promoting ASRH for the next 15 years were established. Unfortunately, despite investments in comprehensive sexuality and HIV education for young people over the past decades, grave trends are still being observed, which continues to point toward the necessity for better sexual health education and services [4].

### 1.1. Sex Education

Sex education refers to “an age-appropriate, culturally relevant approach to teaching about sex and relationships by providing scientifically accurate, realistic, non-judgmental information” [2] (p. 69). This definition acknowledges that the aim of sex education extends beyond the transfer of knowledge on human physiology, reproductive system, or the prevention of STIs. Rather, sex education is conceptualized holistically with the goal of empowering youths to better understand their sexuality and relationships, which will ultimately improve adolescents’ sexual health and overall quality of life [11]. This is in line with WHO’s delineation of sexual health as “a state of physical, emotional, mental and social well-being in relation to sexuality; it is not merely the absence of disease, dysfunction or infirmity. Sexual health requires a positive and respectful approach to sexuality and sexual relationships, as well as the possibility of having pleasurable and safe sexual experiences, free of coercion, discrimination and violence. For sexual health to be attained and maintained, the sexual rights of all persons must be respected, protected, and fulfilled.” [12] (p. 4). 

Generally, sex education focuses on delivering facts about sexual and reproductive health. However, the content, messages, and approaches of delivering sex education vary across countries [13]. In both the West and non-Western Chinese contexts, the implementation of sex education remains a contentious subject in public health and education policy on several grounds. First, it is the deep-rooted perception of sex as a “taboo” itself, especially in the Asian cultures. Some skeptics argue that sex education encourages promiscuity among youths, and believe that this issue should be avoided so as not to “awaken the sleeping bear”. Second, while policy makers, educators, and parents witnessed that adolescent sexual behavior is getting “out of control”, they disagree on how youngsters’ problematic sexual behaviors can be minimized; and third, on whose responsibility it is to control our youths in this area [14]. Sex education programs may be school-based that are led by teachers, social workers, health professionals, or peers; community-based; or family-based. In addition, there are various approaches to sex education including abstinence-only, abstinence-only-until-marriage, abstinence, and comprehensive sex education.

### 1.2. Abstinence-only or Abstinence-only-until-marriage Education Approaches

Abstinence-only education (AOE) or abstinence-only-until-marriage (AOUM) programs, with its religious origins, advocate the complete refraining of sex outside of wedlock, including masturbation. Abstinence is perceived moralistically, where notions of virginity and chastity are highlighted and “teaches that a mutually faithful monogamous relationship in the context of marriage is the expected standard of human sexual activity” [15] (p. 97). Students’ character and morality are core issues. Under this approach, policies prohibit or limit the mention of contraception in sex education, and biased findings of contraceptive methods (e.g., condoms and birth control pills) as failures are often presented. Advocates believe that providing students with information on where to obtain and how to use contraception will undermine the abstinence-only message and encourage immoral and health-compromising sexual behaviors which will, in turn, increase the rates of sexually transmitted diseases (STDs) and unwanted pregnancy [16]. As such, they believe that abstinence-only is the sole unfailing way to prevent STDs and unwanted pregnancies. The implementers of abstinence-only programs discourage sexual activity by employing tactics to instill fear, shame, and guilt in relation to sexual activity. Moreover, it has been criticized that AOE programs present scientifically inaccurate information, use stereotypical gender roles that discriminate against female students (e.g., portraying women as the “gatekeepers” of men’s virginity, blaming women for men’s sexual indiscretions), and overemphasize religious messages [17]. Nevertheless, this approach is mostly welcomed by conservative and religious groups [18].

### 1.3. Abstinence or Stress-abstinence Approach

While AOE and AOUM approaches perceive abstinence under a moral lens, proponents of abstinence approach conceptualize it as a behavioral and public health issue, encompassing behaviors such as delaying sex, never having vaginal sex, or refraining from engaging in further sexual intercourse even if one has already had sexual experience. Abstinence, however, does not include other sex-related behaviors such as petting, kissing, mutual masturbation, oral, and anal sex [19]. 

### 1.4. Comprehensive Sex Education

Under comprehensive sex education (CSE), abstinence is also included in the curriculum. However, in contrast to the aforementioned approaches, abstinence needs not be stressed. Rather, comprehensive sex education incorporates a range of prevention strategies on contraception to prevent STDs and unwanted pregnancy, and highlights the importance of safe sexual practice [19]. According to The International Planned Parenthood Federation, CSE refers to “education about all matters relating to sexuality and its expression. Comprehensive sexuality education covers the same topics as sex education but also includes issues such as relationships, attitudes towards sexuality, sexual roles, gender relations and the social pressures to be sexually active, and it provides information about sexual and reproductive health services. It may also include training in communication and decision-making skills” [20]. 

CSE is an empowerment-based rooted with values and practices emphasizing human rights, gender equality, participative learning, youth advocacy and civic engagements, as well as cultural appropriateness. It aims to equip students with knowledge, values/attitudes, and skills to facilitate students to make informed decisions that promote sexual health [10,21]. Research supports the implementation of CSE. For instance, the United Nations Population Fund (2016) reported that CSE does not lead to earlier sexual debut or risky sexual behaviors which may be a misconception held traditionally [22]. Rather, approximately two in three CSE programmes evaluated showed reductions in risky sexual behaviors. 60% of the CSE programmes yielded positive effects such as increased condom use or reduced teenage pregnancies. In addition to the aforementioned effective outcomes of CSE, the authors firmly believe that healthy sexuality plays a crucial role in holistic positive youth development. Without healthy sexual attitudes and behaviors, adolescent development will be adversely affected.

## 2. Methodology

A comprehensive review on evidence-based sex education programs in developed countries from the West (i.e., the U.S., the UK), and non-Western Chinese regions (i.e., Mainland China, Hong Kong, and Taiwan) was conducted. These countries were selected as they provide a diversity in terms of their political and cultural background, the role that the government plays in sex education policy making, as well as its varying approaches in implementation, training, research, and evaluation. More specifically, despite continuous declines in the past decades and currently at its record lowest, U.S. pregnancy rates remain to be the highest in the developed world (43 per 1000 females in 2013) [23]. While in Europe, the highest adolescent pregnancy rates were reported in England and Wales (47 per 1000 females in 2011) [24]. The U.S. and the UK are also the largest English-speaking countries in the globe. 

Compared to the Netherland which mandated sex education with the second lowest adolescent pregnancy rates in Europe, the education system in the U.S. and UK are far less consistent in terms of their provisions. These inconsistencies therefore warrant more detailed analyses [24]. In a recent Lancet article [25], figures from Asia in 2014 revealed that unintended pregnancy rates per 1000 women aged 15-44 had a 20% decrease since 1990, among which Southeast Asia reported the highest percentage decrease (31%). Mainland China and Taiwan were included in the present study due to their shared deeply entrenched roots of Chinese Confucian culture. Hong Kong serves as a unique region for examination as well, given its intermingled influences from traditional Confucian ideology and Christian values brought along from its former British colonization (from 1842 to 1997) which has been found to impact on citizens’ sexual attitudes [26]. 

For published materials, using a combination of search terms such as sex education, evidenced-based sex education programs, relationships and sexuality education, schools, adolescents, youths, STIs, HIV, or AIDs were used to search for relevant information in the online databases (PsychINFO, PubMed, the Cochrane Central Register of Controlled Trials, Web of Science), Internet searches (Google Scholar, Guttmacher Institute, UNIAIDS, WHO) to identify articles that detailed and evaluated evidence-based sex education programs. In addition, we conducted searches for grey literature using relevant keywords on Google to identify sex education programs and policies led by government bodies or other agencies and organizations relevant to adolescent health.

In the present review, we focused on analyzing the selected regions based on their policy, practice, training, and evaluation; which have been previously identified as key aspects of sex education in schools to ensure that students’ health and wellbeing are maximized [27]. Moreover, in this review, whether a sex education program is effective will be determined by various outcome as outlined by Kirby (1998) [28]. These indicators include (a) enhanced knowledge; (b) changed attitudes (e.g., attitudes towards premarital sex, birth control, STDs, etc.); (c) acquired skills (e.g., making decisions pertaining to sexual relations, being able to communicate feelings about the use of contraception and sexuality); and (d) learned behaviors (e.g., frequency of sex, use of contraception).

The present review contributes to the existing literature in various manners. 

First, up until now, to the best of our knowledge, articles have not been published to paint such a comprehensive picture on sex education of different Chinese societies. Second, this review provides a thorough landscape of sex education in two English-speaking countries with the largest populations in the Western world. Finally, this paper identifies common issues and challenges faced in both the Western and Chinese communities concerning the provision of sex education.

## 3. Sex Education in the United States of America

### 3.1. Policy

In response to the increased prevalence of out-of-marriage pregnancies and the pandemic of HIV/AIDS, sex education has been an important public health policy issue in the U.S. over the past four decades. There was an obvious pressing need for formal education targeted at adolescents on protective health topics such as the use of contraception, and knowledge on sexually transmitted diseases. In the late 1990s, the U.S. government proposed a singular Abstinence Only Until Marriage (AOUM) approach to sex education with up to 49 of the 50 states implementing programs to promote AOUM at schools. However, over the years, empirical evidence pointed toward the lack of effectiveness of AOUM approach in delaying sexual debut or reducing risky sexual behaviors [29]. Thus, under President Obama’s administration, the AOUM approach was proposed to be eliminated and replaced by a more comprehensive programs—one that “normalizes teen sex” [30]. This approach is based on the assumption that it will not be possible to dissuade a certain proportion of the adolescent population from sexual activity. Therefore, the best approach is to teach and promote the use of contraception which may lower the rate of unwanted pregnancy and STDs. Simultaneously, for youths that have yet to become sexually active, continued abstinence is still promoted [31]. Under Obama’s leadership, the proposed budget was increased to support programs such as the Teen Pregnancy Prevention Program, which equipped youths with the necessary skills to ensure lifelong sexual health and wellbeing [30]. Yet a turn was taken under Trump’s administration which reverted back to supporting the abstinence approach, as reflected by the priority of funding programs that promote abstinence, sexual risk avoidance, and provide cessation support (i.e., encouraging sexually active youths to deter having sex) [32]. 

At present, sex education is mandated on a state-level, where different states, districts, and school boards have the autonomy to determine the implementation of federal policies and funds for sex education. Since there is a lack of cohesive and consistent policies governing the implementation, the system has been criticized to be a “highly diverse patchwork of sex education laws and practices” [29] (p. 595). Of the 50 states and the District of Columbia, merely 24 of them have mandated sex education classes in public schools and 34 states mandate HIV education [33]. However, in terms of content, there is great variation across states. For instance, Rhode Island, West Virginia, and the District of Columbia provide a detailed age-appropriate standard on the topics to be covered in sex education. However, the majority of the states such as Kentucky and Nevada offer minimal guidelines as to what should be included in the sex education curriculum [34]. Although comprehensive sex education (CSE) is gaining popularity, and is supported by many organizations, given its effectiveness in delaying sexual activity and decreasing risky sexual behavior among young people have been evidenced [35], the Abstinence-Only Education approach is still adopted by states that are more likely to hold socially and politically conservative beliefs, such as Tennessee and Montana [36].

### 3.2. Practice

In terms of implementation, while every state provides guidance on the content of sex education, individual school districts possess the autonomy to decide on how and when sex education should be taught. In the U.S., sex education is often included as part of health or physical education (PE) curriculum for high school students, which is delivered by health and PE teachers. Unfortunately, [29] up until now, there remains a lack of evidence-based conceptual models on comprehensive sex education for adolescents in the U.S. According to the Center for Disease Control and Prevention (CDC) [37] (p. 3) “Exemplary sexual health education is a systematic, evidence-informed approach to sexual health education that includes the use of grade-specific, evidence-based interventions”.

Indeed, efforts have been dedicated to developing guidelines for the implementation of sex education. In 2014, the CDC [37]“proposed 16 critical topics that should be included in sex education as a part of the School Health Profile, including (1) How to create and sustain healthy and respectful relationships; (2) Influences of family, peers, media, technology and other factors on sexual risk behavior; (3) Benefits of being sexually abstinent; (4) Efficacy of condoms; (5) Importance of using condoms consistently and correctly; (6) Importance of using a condom at the same time as another form of contraception to prevent both STDs and pregnancy; (7) How to obtain condoms; (8) How to correctly use a condom; (9) Communication and negotiation skills; (10) Goal-setting and decision-making skills; (11) How HIV and other STDs are transmitted; (12) Health consequences of HIV, other STDs and pregnancy; (13) Influencing and supporting others to avoid or reduce sexual risk behaviors; (14) Importance of limiting the number of sexual partners; (15) How to access valid and reliable information, products and services related to HIV, STDs, and pregnancy; and (16) Preventive care that is necessary to maintain reproductive and sexual health” (p. 1). However, a nationwide survey across 48 states revealed that less than half of the high schools and merely one-fifth of middle schools cover all of the 16 proposed essential sex education topics. Moreover, within a school year, an average of merely 6.2 hours of instruction was dedicated to human sexuality, 4 hours or less on HIV, STDs, and pregnancy prevention [37]. 

Despite efforts to promote adolescent sexual health, teen pregnancy rates in the U.S. continues to be ranked as the highest among Western countries. This phenomenon has been attributed to the resistance towards the incorporation of a standardized sex education curriculum across districts. A report published by the Guttmacher Institute [38] illuminated on this disparate implementation where it was found that merely 26 states mandate that the content of instruction be age-appropriate, 13 states to be medically accurate, eight that the materials must be free from race or gender bias, eight that the content must include information on sexual orientation, and two that it should be religion-neutral. The lack of consensus across districts on the approach and content of sex education may be due to various reasons, including individuals’ attendance at religious services and political orientation [39].

### 3.3. Training

The effectiveness of school-based education programs depends highly on teachers. Studies have shown that instructors’ commitment to, as well as comfort with the delivering of sex education impacted on ones’ teaching ability [40]. The positive relation between teachers training and implementation fidelity has been documented. Teacher workshops on sex education should provide strong justification, knowledge, and skills for program delivery, enhance commitment and support to the program, and underscore the significance of program fidelity to teachers [41].

In the 2018 Sex Ed State Legislative Mid-Year Report [42], 109 bills across 27 states were introduced or carried forth in hopes of advancing sex education instruction in schools; 48 of the bills were related to teacher training requirements. Although there were few specific guidelines regarding teachers’ training, it was required that the State Department of Education or other organizations set minimum training criteria. The National Teacher Preparation Standards for Sexuality Education was developed to provide guidance for educators teaching sex education in middle and high schools. A total of seven standards with reference to: (1) professional character; (2) diversity and equity; (3) knowledge on materials; (4) legal and professional ethics; (5) preparation; (6) implementation; and (7) assessment were outlined. In addition to training, CDC also noted that it was critical to provide educators with materials needed to effectively teach sexual health topics [37], as well as strong support from administration, and ongoing technical assistance [41].

### 3.4. Evaluation and Research

Findings from evaluation studies on the effectiveness of different sex education approaches have been mixed. While some reviews and meta-analyses provided support to comprehensive sexual risk reduction programs in reducing targeted sexual risk behaviors; studies suggest otherwise [10,43]. In an analysis consisting of 60 studies examining the impact of school-based comprehensive sex education, Weed and Ericksen [31] found that CSE was not effective to decrease teen pregnancy as well as STD rates and to increase abstinence and condom use at 12-months post-program [16]. Several studies were identified to have yielded negative effects including higher levels of sexual initiation, oral sex, and reduced contraceptive use [44,45]. On the other hand, some school-based abstinence education programs revealed sustained increases in teen abstinence. 

In order to gain an understanding of the effectiveness of the implementation of sex education, CDC [46] highlighted the importance of evaluating program outcomes. Due to the inconclusive results obtained from studies evaluating the effectiveness of programs adopting various approaches in promoting adolescent sexual health, the Institute for Research and Evaluation called for more evaluation studies using rigorous methodologies and meaningful indicators (e.g., sustained post-program effects), as well as replication studies to verify positive findings of existing sex education programs which would better inform public policy [31]. This was echoed by Chandra-Mouli, Lane, and Wong [47] who recommended “greater attention to the adaptation of evidence-based prevention science approaches that simultaneously address risk and protective factors… This should include the creation of a database that documents best and promising practices in prevention science and adolescent health” [47] (p. 339). Please refer to Table 1 for a systematic presentation of the sex education initiatives in the U.S.

## 4. Sex Education in the United Kingdom

### 4.1. Policy

In the 1960s, sex and relationship education (SRE) was introduced as a part of the UK school curriculum. However, because of diverging sociopolitical ideologies and the absence of consensus among stakeholders (e.g., parents, religious groups), the objectives of sex education remain unclear. Particularly, a contention lies in whether health or moral values should be primarily addressed [48]. In the 1980s, the Conservative government instilled traditional sexual values through moral teachings. By the late 1980s, sex education was intended to ensure social stability by addressing public health issues such as the rising prevalence of unplanned pregnancies, STIs, and HIV/AIDS. This resulted in the passing of legislation which was largely prohibitive, such as Section 28 of the Local Government Act in 1988 which banned the “promotion” of same-sex relationships in schools.

While there is no standardized curriculum for SRE in the UK, progressive developments were made in 2000, when the Department of Education and Employment published a statutory Sex and Relationship Education Guidance which provided guidelines for SRE within schools where the importance of respecting social, cultural, and sexual diversity was highlighted: “young people, whatever their developing sexuality, need to feel that sex and relationships education is relevant to them and sensitive to their needs” [49] (p. 12). Under legislation passed in 2017, relationship education is compulsory in all primary schools. Whereas, SRE must be a part of the secondary school curricula [20]. Earlier in 2018, the Government announced that sex education will become compulsory for school children from September 2020 and is currently consulting stakeholders including parents, subject matter experts, and youngsters on the content of the curriculum [50].

### 4.2. Practice

At present, there is no standardized SRE curriculum for schools which implies that schools have the autonomy to develop their own programs to cater to their respective students. Taken as a whole, UK sex education places sexual intercourse within the contexts of marriage and fidelity [51] and emphasizes abstinence. SRE can be categorized into five types, namely: (1) sexual abstinence-only programs; (2) comprehensive programs; (3) pregnancy-prevention programs; (4) HIV-prevention programs; and (5) school-based or school-linked sexual health services (e.g., primary care clinics, youth service drop-in facilities, and outreach services which provide contraception and sexual health support or advice) [52]. Although there is no standardized curriculum or program, the government has set out a number of broad requirements that every state-funded school must adhere to when implementing SRE [49]. Since SRE is delivered within the Personal, Social and Health Education (PSHE) framework and its content is determined at the school level, the content of the SRE programs are heavily influenced by localized district factors including prevalence rates of unwanted pregnancy, STIs, parents’ needs, etc. Some counties dictate what SRE should contain, but often individual teachers are left to decide on the approach and method of implementation [51]. 

This self-governing arrangement has been prized by stakeholders including parents, governors, and school management. However, experts have criticized that this value-led approach merely reflects the interests and principles of stakeholders, while overlooking the actual needs and wellbeing of youths [53]. The content commonly found in most programs includes knowledge on HIV and AIDS, contraception, methods to prevent STIs, as well as risks and consequences of unprotected sex, pregnancy, STIs, and reproductive health [52]. These topics heavily focus on biological aspects covering topics such as puberty and sexual reproduction, spread of viruses, etc. with the aim of delaying early sexual activity and reducing sexual partners, and encouraging contraceptive use. Youths are deprived of certain knowledge about sex and sexuality [54]. Specifically, present guidelines on SRE fails to include contemporary issues such as sexting, internet pornography, cyberbullying, or LGBTQ identities, and the notion of consent [20]. Moreover, students reported that they felt uncomfortable having their own teachers teach them about sex due to blurred boundaries and a lack of anonymity [55].

In addition to school-based efforts, members of the wider community also play crucial roles in the provision of SRE for youths. For instance, some schools work with health professionals such as doctors and nurses in the development and delivering of SRE programs. Youth workers also play important roles in outreach work to provide confidential advisory services to children and young people outside of the school context [49]. 

### 4.3. Training

Evidently, the effectiveness of school-based SRE relies predominantly on teachers. Yet, students reported dislike of their own teachers delivering SRE as they sensed the teachers being embarrassed and were poorly trained in this aspect [55]. It can be difficult “… to discuss sex, particularly when the discussion is led by untrained teachers who are not given sufficient help to deliver the material, and who as a result may be uncomfortable talking about it” [56]. Indeed, teachers themselves have reported feelings of awkwardness when delivering SRE [57,58]. Up to 80% of teachers were not confident and perceived inadequately trained on SRE. 

When teachers were asked about the barriers they faced having to implement SRE, about half of the interviewed teachers identified the lack of training, and lack of time to develop and coordinate the SRE programs. Fortunately, teachers do receive training or support from external agencies in relation to the delivery of SRE. These workshops were usually coordinated by the local education authority and took place at local hospitals where teachers are given the opportunity to work together and exchange ideas with health professionals such as doctors and nurses on sex education [59]. In terms of resources support, merely 9% of the teachers found the materials and resources provided to be useful to their SRE teaching. Approximately, one in four teachers believed that the current SRE fails to prepare children for the future [60]. This further highlights the pressing need for the development and implementation of effective SRE teachers training. For example, an evaluation study was conducted on an in-service program for training SRE teachers to deliver a sex education program entitled “SHARE”. Participants of the teacher training program found it to be highly beneficial. Particularly, teacher participants received social support from colleagues which they found to be valuable. The training component also enabled teachers to be familiarized with the teaching resources which helped to boost their confidence in delivering SRE [61].

Finally, one of the main criticisms of the existing SRE is its heterosexist orientation which highlights the importance for teachers to reflect on different aspects of SRE practice, update their knowledge on sex and sexuality through attending training and workshops. Specifically, it was recommended that training should equip teachers with knowledge and skills that would enable the development and implementation of up-to-date curriculum that takes into account youngsters’ sexual identities, relationships, and cultural backgrounds [62].

### 4.4. Evaluation and Research

According to the National Survey of Sexual Attitudes and Lifestyles [56], young people’s sexual practices have changed over the last 20 years. The proportion of sexually active 16 to 26 year olds who reported having had sexual intercourse with opposite-sex partners during the previous year increased from one in 10 females and one in 10 males in 1990–1991, to one in five females and one in four males in 2010–2012. These figures call for the pressing need for SRE in schools, families, and the community. Wight [63] conducted a review of evaluations on three nationwide large-scale comprehensive sex education programs (i.e., SHARE, RIPPLE, and HEALTHY RESPECT) implemented in the UK. The sample included over 22,000 students from nearly 80 schools. It was found that all three programs helped to enhance students’ sexual health knowledge and certain attitudes. However, findings revealed that the programs did not yield remarkable improvements in adolescents’ sexual health outcomes. 

Using a meta-ethnographic method reviewing 55 publications mainly from the UK, the current SRE was criticized for its lack of statutory status, outdated government guidance and the observation that one-third of UK schools delivered unsatisfactory SRE [55]. These problems are attributed to two main reasons. First, schools overlooked the emotional laden and unique nature of sexuality. As a result, the curriculum was taught in a way similar to that of any other academic subjects. Second, there is a reluctance to accept that sexual activity is high in some adolescents. This results in a discrepancy between what is taught and what students are experiencing [55]. Moreover, the current SRE content fails to address contemporary sexuality issues. For instance, over 50% of lesbian, gay, and bisexual youngsters reported that issues surrounding non-heterosexual relationships have not been taught in their schools. Similarly, 85% of students shared that SRE education did not include biological or physical aspects of homosexual relationships [64]. Researchers pointed out that more research must be conducted on same-sex attitudes and sexual behaviors among youngsters which will guide education policy to safeguard and enhance the health and well-being of youths. See Table 1 for a systematic presentation of the sex education initiatives in the UK.

In the following, sex education in three Chinese societies, including Hong Kong, Mainland China, and Taiwan will be reviewed with reference to policy, practice, training, and evaluation as well as research. (Please refer to Table 2). They are included because they are under the strong influence of Chinese culture and social thoughts, such as Confucianism.

## 5. Sex Education in Hong Kong

### 5.1. Policy

The Family Planning Association of Hong Kong (FPAHK) began to promote sex education in Hong Kong in the 1960s, focusing on family planning and contraceptive knowledge [65]. Until 1971, the memorandum issued by the Education Department (now Education Bureau) then suggested including topics of sex education in some formal subjects in all Hong Kong schools [5]. In 1986, the Education Department published a more detailed guideline on sexuality education in secondary schools with recommendations on topics, resources, and references for promoting relevant programs. This set of the guideline was revised further in 1997 for strengthening the promotion, but it has not been revised since then [5].

According to the guidelines formulated in 1997, sexuality education includes five key concepts, including “human development, health and behavior, interpersonal relationships, marriage and family, and society and culture” [5] (p. 23). Unfortunately, this framework is for reference only. In 2000, the Education Department integrated sexuality education into the curriculum of Moral and Civic Education, and revised its framework in 2008, with the purpose of assisting schools in implementing sexuality education systematically. One problem of this curriculum is that it lacks a well-articulated conceptual framework. For example, while psychosocial development such as positive youth development shapes the sexual and reproductive health of adolescent [66] the proposed curriculum just focuses on the social and sexual relationship in a shallow manner without covering psychosocial development such as emotional competence and moral competence. 

### 5.2. Practice

Regarding the implementation of sexuality education in schools, it is suggested by the Government to name it as “life education”, especially for junior students [67]. Teachers are also assigned to take up sex education that covered wider topics using various teaching resources and learning activities [68] (p. 90). Nearly all schools in Fok’s survey reported that sex education is provided by adopting the comprehensive approach that aims at preparing students for expressing their sexuality appropriately, but not just focusing on the prevention of negative consequences of casual sex. However, Lee [2005] argued that most schools still passively rely on school social workers, community resources and NGOs in carrying out sexuality education [65]. 

The Government findings showed that 72% of the 134 interviewed schools provided “life-skills based” AIDS or sex education in the 2011/12 school year [5]. For others, about 67% and 46% of the interviewed schools arranged an average of only three hours for each academic level, by relying on the programs of NGOs and the Department of Health respectively per year [5]. In the implementation, prevention of HIV had been mentioned by 60% of interviewed schools, and the use of condom had been taught by about 80% of interviewed schools via multiple learning activities or programs [5]. Besides “life-skills based” programs, 86% of the interviewed schools spent around 4 hours to provide AIDS or sex education in the main subjects, and 28% used about 3 hours to provide relevant information in the life-wide learning activities [5]. However, there is a lack of a systematic database recording diverse sex education programs in schools. As a result, the schools might have difficulties and low incentive to adhere to an evidence-based sex education program. Most importantly, evidence-based programs on sex education for schools do not exist in Hong Kong.

### 5.3. Training

As for teachers’ training, it is revealed that the training programs of sexuality education for teachers are usually short-term, scattered, and without clear objectives [65]. As reported by the government, only 66% of teachers had received training on AIDS, sex or life-skills based education. The training sessions were provided in the form of professional development programs by the Education Bureau, training programs by the Department of Health or NGOs, or simply materials from the Internet [5]. In the findings, only 4.1 teachers in one school on average had received relevant training since they had been working in their schools, and about 2.1 of them had taught sex education topics in the last school year. At the same time, a mean of 4.9 teachers per school had taught relevant topics without attending any relevant training program [5]. Besides, roughly nine-tenths of the 198 secondary schools under study expressed that they lacked trained teachers for teaching sexuality education [65,69]. At the same time, collaboration with multiple disciplines is also rare. Lee pointed out that teachers and schools could gain more inspirations from working with other professionals like clinical practitioners in conducting sex education in schools [65]. 

### 5.4. Evaluation and Research

Concerning the evaluation of sexuality education in Hong Kong, the former Education Department had carried out an investigation on the sexuality education implementation in secondary schools in 1987, 1990, and 1994. The findings showed that most schools had difficulties in the implementation [65]. In 2012–2013, the Government further conducted a territory-wide survey which aimed to understand the implementation of life skills-based curriculum in the junior secondary schools, especially on HIV/AIDS and sex topics [5]. Besides, several NGOs and research groups had conducted multiple types of research. For example, a survey regarding the implementation was conducted by the research centre of the Hong Kong Institution of Education in 2001 [65,69]. In 2016, the Family Planning Association of Hong Kong and the Aids Concern also reported that more young people have engaged in sexual activity with insufficient information and support from school-based sexuality education [6,70]. Even though findings from the Government and NGOs actually indicated the sexuality education in Hong Kong have to be strengthened, continuous and specific evaluations of the Government and schools are inadequate. With the lack of regular research and assessment, the effectiveness of sex education programs in Hong Kong remains unknown.

## 6. Sex Education in Mainland China

### 6.1. Policy

In mainland China, the development of sex education began in the early Republican period. During the 1920s, it was believed that the population was a key source that would help China to become a strong and rich country, so it should be carefully measured and monitored by the State. Links between modernization and “issues of sex, reproduction, women’s liberation and eugenics” were developed [71] (p. 533). Until the People’s Republic of China (PRC) established in 1949, the new Communist leadership regarded “eugenics, genetics and physical anthropology as ‘bourgeois science’ that should be criticized” severely, and sexuality was an area under the control of the State [71] (p. 534). In the late 1950s, the Government introduced birth control in the curricular of middle schools. In the 1950s to 1960s, sex education was perceived as a vital part in sexual physiology. In 1963, the Government declared the necessity of promoting scientific sexual knowledge among young people, where sex education was stressed as an essential element in a healthy growth of the Chinese population [71]. However, the sex education in China was once paused during the Cultural Revolution as sex was banned from all aspects [71]. 

After the Cultural Revolution in the late 1970s, the One Child Policy and a shifted focus on the quality of the population instead of quantity were proposed. The topics related to “birth control, eugenics and sex education” were brought back to the debates, and The State Family Planning Commission also added sex education in the agenda of the 7th Five Year Plan (1986–1990) and the 9th Five Year Plan (1995–2000) [71] (p. 535). Then, the first school-based health education policy with guidelines listed was carried out by the Government in both primary and secondary schools in 1992 [71]. The China’s Ministry of Education further revised it in 2003 and 2008 [72]. It is noticed that sex education of China has long been guided by the developmental direction of the nation, instead of any theoretical model. This influenced the practice in schools. 

### 6.2. Practice

In the early 1920s, school-based sex education was only perceived as a supplement at that time [71,73]. After the announcement of birth control policy in the 1950s, sex education became mandatory in schools. Then “the State Education Commission and the State Family Planning Commission jointly issued the ‘Notification on the Development of Adolescent Education in the Middle Schools’ in 1988”, which announced schools should take the major role in sex education and formally integrated it into the middle school curricula all over the country [71] (p. 537). The abstinence-based approach was adopted and “sexual physiology, sexual psychology, sexual morality, and socialist moral education” were the foci [71] (p. 537). In view of earlier sexual maturation of adolescents in 1990s, the focus shifted to more life skill-based that issues related to premarital sex, HIV/AIDS, and unwanted pregnancies were incorporated in the Health Education of the secondary schools and universities [71,74]. Although the youths could be granted with limited sexual rights and responsibilities in the current practice, prevention of STIs and importance of contraception are not clearly stated in the guidelines [72]. Apart from the insufficiency in the guidelines, evidence-based sex education programs and relevant database are also lacking in guiding the practice in China. The effectiveness of the current practice is indeed found to be unsatisfactory [72].

### 6.3. Training

Improving teachers’ training course on sex education was one of the main objectives in the Notification published by the State Education Commission and the State Family Planning Commission in 1988 [71]. Although some training programs were provided to part of the teachers in previous years, when it comes to the topic of safe sex education, it created discomfort in most teachers as nearly all training programs stem from the abstinence-based approach [71]. This issue still remains unsolved although efforts in interdisciplinary collaboration have been made. For example, in the “International Conference on Sexual Health Education for Youth in China” held in 2005, professionals such as teachers, doctors, scholars, and social workers gathered and discussed the pressing issues of sex education in China [71] (p. 539). Other than the content covered in the training, cultural sensitivity is also a critical issue to work on. 

### 6.4. Evaluation and Research

Scientific works on sex education were noticed since the 1920s. Zhang’s “Sex Histories” published in 1926 is regarded as the first scientific work in China that systematically gathered informants’ sexual experiences plus his suggestions on sex education [71]. In fact, many scholars have conducted various research on sex education in China with its growing debates in society. These findings not only encourage further evaluation and research in sex education, but also provide the Government with more information to review the current implementation. In fact, several studies in the 1980s showed that the Government recognized the importance of schools in providing information on birth control [71]. However, in contrast to the numerous studies done by the scholars and different organizations such as the UNESCO, official evaluation conducted by the Government on the mandatory sex education programs and training appears to be inadequate. 

## 7. Sex Education in Taiwan

### 7.1. Policy

After the Chinese Civil War in 1949, sex education was introduced in Taiwan, where the education system was strictly guided by the Government and legislation [75]. There are three stages of development to sex education in Taiwan from initiation, developing, to integration [76,77]. The initiation stage refers to the period from 1960 to 1988. Due to the announcement of the Guide for Policy on Population in 1969, birth control was started via the practice of population education in Taiwan. Starting from 1972, content regarding sex education was greatly added in different subjects like Health Education and Biology [77], and population education was implemented in all primary as well as secondary schools in 1983 in order to promote the Government policy [77,78]. From 1989, sex education in Taiwan moved on to the developing stage after several non-Governmental organizations had been established. In this period, conferences on sex education were held and social movement and research aiming at gender equality were also initiated, which indicated that public awareness of sexual health issues was growing [77,78]. In 1991, The Department of Health and the Ministry of Education began to collaborate in promoting sex education in schools, with the new focus on promotion of “understanding the harmonious relationship between genders” [77] (p. 35). In 1997, sex education stepped forward to the integration stage with the help of the education reform policy) [79]. The Ministry of Education introduced “The Nine-Year Joint Curriculum” in 1998, where gender education became a key teaching element in solving gender inequality [77] (p. 35). The implementation of education reform policy made a significant impact on sex education in Taiwan. 

### 7.2. Practice

In the National Curriculum Standard, all subjects had statutory status and textbooks were all published by the Government agencies. After the introduction of the Nine-Year Joint Curriculum, the status of all the learning areas remained unchanged, but the schools could have more autonomy in the implementation. Under Government monitoring, the teaching materials still adhered to the Government guidelines systematically [77]. Teachers are provided with relevant studies and practical information on the policy to guide their practice [77]. Different ideologies in sex education like sexual liberation, gender issues and health education were included [76,77]. Tu [77,80] noticed that the content in the new curriculum is richer especially on topics related to the social relationship than before, and the condition was similar across different areas in Taiwan. It is believed that the statutory status of sex education and the clear guidelines provided by the Government contributed to such uniformity in practice [77,81]. More specifically, it is found that most teachers adopted lecturing as the main way to teach sex education, with the assistance of multimedia and occasional demonstration [77,82]. However, students indeed showed more interests in the additional and non-traditional methods [77,83]. This might reflect an inadequate investigation into the pedagogy of sex education, as compared with the emphasis on its knowledge and information [77]. Therefore, though sex education is implemented in all primary and secondary schools in Taiwan with clear suggested topics, the teaching methods require more reflection.

### 7.3. Training

In order to ensure the teachers are qualified to conduct sex education programs, a lot of training, conferences and programs have been provided by the Government. Besides promoting gender education, theoretical knowledge and practical skills are included in teachers’ training programs at universities in the four-year institutional training. The graduates would have to complete a half-year field practice before officially recognized as a qualified teacher. The “Teacher Act” in 1995 led to more teacher training programs provided in the Taiwanese universities [77]. The Ministry of Education also authorizes the non-Governmental organizations to conduct additional training programs, talks, conferences and events for schools, professionals, as well as the public on sex education since the 1990s [75]. These organizations promote interdisciplinary communication by inviting members and collaboration from different backgrounds and professions. Teachers agreed that their “development of educational ideology and theories, professionalism and knowledge of sex education” [77] (p. 42) are strengthened with ongoing and additional training [84]. While the ongoing training is effective, Yu [77,85] realized that the participation rate in the training could be promoted by rearranging the training schedule. Instead of having the training during school time, teachers expressed that they would like to have the training during school holidays more [77,82].

### 7.4. Evaluation and Research

It was claimed that sex education in Taiwan is evidence-based in nature [77] and the research can be found in specific databases. From the information captured from 1979 to 2004, it was found that research on sex education had significantly increased. For example, the total amount of relevant research in the three databases was only four in 1979–1984, but it greatly increased to 103 in 2000–2004 [75]. Apart from the research projects commissioned by the Department of Health, there are also heaps of research to evaluate the effectiveness and impact of the implemented sex education conducted by different scholars and organizations [75]. For instance, Yen and colleagues [77,83,86] found that sex education improved students’ knowledge on sexuality, and extending the time and extra activities for sex education could bring more impacts to their knowledge and attitude. This influenced the development of the teaching approach on sex education as the findings in research would be used to provide advice for the Government [77]. Regardless of the diverse comments on the research, the need for sex education is consistently recognized by multiple parties in Taiwan. 

## 8. Discussion

The purpose of this paper is to outline the school-based sex education policies and programs in three Chinese-speaking societies in Asia and two English-speaking countries in the Western context. There are several unique features of this review. First, the review can enable researchers to understand school-based sex education in more individualistic societies (United States and the UK) and collectivistic societies (three Chinese societies in Asia). In the Chinese culture, collectivistic interests such as social stability and family harmony are emphasized. As individual sexuality such as free sex may pose a threat to social order and family harmony, sexuality is commonly seen in an inhibitory manner in traditional Chinese cultures. Second, in view of the rapid urbanization and Westernization in different Asian societies, it would be theoretically and practically to know what school-based sex education is implemented in Chinese societies and the content of such programs. Third, several Chinese societies including mainland China, Hong Kong, and Taiwan were studied which can give a comprehensive view of school-based sex education in multiple Chinese communities. This is essential because Chinese people roughly constitute one-fifth of the world’s population [87,88]. Finally, this is the first scientific study which attempts to review school-based sex education policies and programs in Chinese societies in contrast to two largest English speaking societies.

Several observations can be highlighted from the present review. First, different policies are implemented in different societies under study. For example, while comprehensive sex education covering contraception and safe sex is implemented in some states in the United States, abstinence-only and abstinence-plus programs are implemented in other states of the US. In Chinese societies, different policies are implemented in different places. For instance, while conservative coverage of sexual issues is covered in school programs in mainland China, Taiwan is relatively more liberal. Topics such as the involvement in premarital sex and the use of contraceptive methods are covered in Taiwan high school sex education [89].

The second observation is that theories and scientific findings are seldom taken into account when formulating policies on school-based sex education (i.e., lack of evidence-based policies). For example, the sex education policy in Hong Kong is rather atheoretical, as the practice of letting schools design their “home-baked” sex education program is not evidence-based. In the contemporary literature on positive youth development, it is commonly proposed that psychosocial competencies are an important protective factor for adolescent risk behavior, including health-compromising sexual behavior [90,91]. In other words, with the development of psychosocial competencies such as resilience, emotional competence, connectedness, moral competence, and positive identity such as self-esteem, adolescents would not easily engage in unhealthy sexual behavior. However, sex education usually focuses on knowledge and attitude without making reference to these foundational psychosocial competencies. Another example is that the adoption of the “diffusion” approach is also not supported by empirical evidence. This observation is not surprising because school-based sex education policy-makers who are relatively distant from research and practice of sex education. In the Western context, there is also the criticism that school-based sex education lacks well-articulated theoretical frameworks and robust research evidence. For instance, as mentioned earlier, the U.S. Institute for Research and Evaluation has urged for evaluations adopting more meticulous and clearly articulated methods and indicators, so that the public policy could be refined consistently [31].

Third, there is a dearth of evidence-based sex-education programs, particularly in the Chinese contexts. The existing practice is that schools are “baking” their own school-based sex education program which lacks empirical support. Logically speaking, there can be four types of school-based education programs: (a) programs with negative effect; (b) programs with no effect; (c) programs with unclear effects; (d) programs with a potentially positive effect (i.e., programs with promise); and (e) programs with positive effects. As evaluation is not emphasized in Chinese societies, it can be concluded that school-based sex education programs are basically programs with unclear effects. Most of the time, sex education are window dressing and make the policy-makers and service providers feel contended. As sex education influences the attitude and practice of adolescents, there is a strong need to ascertain whether existing programs would create unintended negative impacts on adolescents [92]. 

Fourth, there is a lack of databases describing and evaluating validated sex education programs in different Asian societies. In North America, there are several databases which provide useful information on different sex education programs. These include the Cochrane database, Campbell Collaboration, MEASURE Evaluation, and Evidence-Based Practices Resource Center etc. For example, the Evidence-Based Practices Resource Center was launched in 2018 by the U.S. government, aiming at providing reliable information and scientifically-based resources for the public, policymakers and the field to improve the practice [93]. These databases with information on validity and effectiveness will enable stakeholders to understand the details of the available programs in the field, and inform practitioners on what “works” or what “does not work”. Stakeholders may then make use of this valuable information to develop or revise existing programs to cater to the needs of their students. This method of knowledge management may help to save resources and redundant overlaps. A similar recommendation has been made to develop such databases in the social work field [94].

Fifth, in Chinese societies, there is a lack of multi-disciplinary collaboration in designing school-based sex education and programs. As there are different dimensions underlying adolescent sexuality, such as the anatomical, physiological, hormonal, physical, cognitive, social, cultural and spiritual dimensions, different professionals have different views on school-based sex education. The different professionals include teachers, principals, social workers, youth workers, counselors, clinical psychologists, pediatricians, health workers, nurses and religious workers. For example, while social workers may adopt a more liberal perspective in implementing school-based sex-education, religious teachers would have great hesitation to teach the knowledge on sexual intercourse and contraception methods. Hence, engagement of different professionals in the process can help to create consensus and “buy-in” and foster multi-disciplinary collaboration. Besides, as students and parents are the major stakeholders, they should be invited in the design of school-based sex education policy and programs. This is important because parents and adolescents commonly have different views on the necessity of implementing school-based sex education and what should be taught. In some places such as the United Kingdom and Singapore, parents may request that their children not to participate in school-based sex education programs. On the other hand, such provision is not present in societies such as Hong Kong and mainland China. 

Sixth, despite the importance of school-based sex education programs, there are no systematic and validated training programs for teachers and allied professionals on sex education. With systematic and validated programs, it is not clear whether the teachers are professional and passionate enough to implement the related sex education programs. Essentially, several areas should be covered in training programs for the potential implementers of school-based sex education programs which include knowledge, attitude, value and behavior in adolescent sexuality with reference to the specific cultural context. Besides, sex education teachers should be familiar with the arguments for and against different positions of sex education (e.g., abstinence versus comprehensive sex education programs). They should also understand how different pedagogical factors and process variables influence the impact of sex education programs in the school context. In the area of positive youth development, Shek and his associates argued that training programs are very important for program success [95].

Finally, while there are many studies on adolescent sexuality, there are comparatively fewer studies examining the factors influencing teaching and learning process and outcomes in sex education. Based on the 5P model, it is recommended that future research should examine how program, people, process, policy and place would influence the outcomes of school-based sex education program. Besides research on teaching and learning, it is necessary to conduct more evaluation on the effectiveness of school-based sex education programs. There is a need to understand the impact of school-based sex education programs over time which is severely lacking in the scientific literature. There is also a shortage of randomized controlled trials and longitudinal evaluation studies to examine the effectiveness of different modes of school-based sex education programs. Besides quantitative evaluation studies, qualitative studies should also be adopted to collect the subjective experiences of program participants and program implementers of school-based sex education programs. This is in line with the spirit of utilization-focused evaluation paradigm [96].

As a limitation of this review, we acknowledge that the literature included in this article remains non-exhaustive. For example, we are aware that there are other European countries such as Netherlands, France, and Australia that adopt a pragmatic approach to sex-positive government policies which have been shown to be more effective than programs in the U.S. and UK. Owing to the “relative” ineffectiveness of sex education programs in the US and the UK, as well as Hong Kong, Mainland China, and Taiwan, we propose further effort should be made to identify the success factors in these countries. Besides, future studies may be conducted in a more systematic manner (e.g., in accordance with the PRISMA guidelines). 

Also, we are aware that teenage pregnancy rates in the Mainland China may be underrepresented due to the high abortion rates. In a recent study of 2370 Chinese adolescents (aged 13–19), 39% reported that they have undergone repeated abortions, and 9% had three or more abortions [97]. As aforementioned, we observed that existing research in the field of sex education often lack methodological rigor and thus is unable to provide conclusive evidence on programs’ effectiveness. Despite the limitations, important implications for policy and practice as well as suggestions for future research were put forth.

## 9. Conclusions

Evidently, worrying trends in sexual wellbeing of adolescents are observed globally with increasing prevalence rates of teenage pregnancy in certain regions and STIs which urged scholars, practitioners, policy makers, parents and young people to examine the implementation and effectiveness of sex education targeted at youths. In addition, the call for stronger Government involvement in promoting sex education in young people can be seen in the 1994 International Conference on Population and Development’s (ICPD) Programme of Action. Against this background, the present review provides an overview of the policy, practice, training, and evaluation and research on sex education in the two largest English speaking countries as well as three Chinese speaking societies. The review shows that there are many gaps and inadequacies in sex education in the places under review. Given the importance of sex education, it is advised that more efforts and actions are required. Particularly, sex education policies and programs should be developed based on scientifically evidence-based theories related to contemporary adolescent development theories and ecological models. Moreover, there is a dire need to equip implementers (e.g., teachers and social workers), as well as parents with the necessary skills to enhance the effectiveness of sex education programs. In addition, in order to gain a more informed perspective as to which factors contribute to program effectiveness, methodologically rigorous evaluation studies adopting both quantitative and qualitative methodologies using longitudinal designs should be employed. Also, databases containing effective programs and measures should be established for more effective dissemination of informed practice. Finally, to promote sexual wellbeing among adolescents in today’s contemporary society, program implementers should take into consideration the complexities of sexual development during adolescence and include topics such as gender, diversity, relationships, empowerment, and consent into existing curricula, rather than merely focusing on the biological aspects of reproduction. In particular, strengthening psychosocial competence in young people may protect them from risky sexual behaviors.

## Figures and Tables

**Table 1:** Summary of sex education aspects in the two large English-speaking societies.

Societies Under Review	Current Policy/Guideline on Sexuality EDUCATION	Program and Its Main Objectives	Actual Practice in Schools	Teacher Training	Evaluation and Research
**United States of America**	Sex education under jurisdiction of individual states Sex education policies are volatile and revised depending on the states’ administrators 2016: One of the most progressive states California passed a law to mandate comprehensive sex education in public schools	Sex education is often included as part of health or physical education (PE) curriculum in public schools Objectives of sex education differs with respects to the approach adopted by the state The overachieving objective of sexuality education includes: Providing accurate information about human sexuality (e.g., anatomy, human reproduction, gender identity, STIs, sexual abuse, HIV/AIDs, etc.) Helping youths develop healthy attitudes, values, and insights regarding human sexuality Equipping youths with communication, assertiveness, decision-making, non-coercive in relationships Encouraging adolescents to make responsible choices by practicing abstinence, postponing sex, practicing safe sex.	Sex education is commonly delivered by health and PE teachers Disparate implementation due to state-level policy Few evidence-based prevention programs exist. In addition to public schools, NGOS also offer community-based sex education	Forms of training: Formal lessons, teacher workshops, talks, online resources Offered by: State Department of Education; Sexuality Information and Education Council of the US; Planned Parenthood; Advocates for Youth, and other NGOs Nature: Follows the teacher-preparation standards proposed by the Future of Sex Education Initiative Performance Assessment Tool for Teacher Candidates Teaching Sexuality Education was developed	Numerous sex education programs have been evaluated and published Evaluation studies with rigorous methodologies and sustained post-program effects were conducted A strong culture of evaluation shaped by researchers and different professionals
**United Kingdom**	2017: legislation passed to mandate relationship and sex education for all school children commencing September 2020	Sex and Relationship Education Guidance was developed in 2000 The Personal, Social and Health Education PSHE objectives include: Helping pupils develop skills to live confidently and independently helping students deal with moral and social questions highlighting the importance of marriage for family life teaching students to understand and respect oneself and others learning the reasons for delaying sexual activity and its benefits Guidelines for sex education has not changed since 2000 Contemporary sexuality issues are often neglected in current SRE programs	SRE is delivered within the PSHE framework No standardized SRE curriculum for schools and are heavily influenced by localized district factors Government has set out broad requirements that state-funded school must adhere to when implementing SRE	Forms of training: Workshops with opportunities for exchanges with health professionals, in-service program training through lectures, forums Offered by: Local education authorities and hospitals; NGOs Nature: Rather piecemeal Teachers reported insufficient training in the delivering of sex education More up-to-date knowledge and skills on contemporary sexuality issues needed	Nationwide large-scale sex education programs have been systematically evaluated using mixed methods and published More research on contemporary issues surrounding sexuality is needed

**Table 2:** Summary of sex education aspects in the three Chinese-speaking societies.

Societies Under Review	Current Policy/Guideline on Sexuality Education	Program and Its Main Objectives	Actual Practice in Schools	Teacher Training	Evaluation and Research
**Hong Kong**	1997: Guideline on sexuality education Policy not updated for almost two decades	Sexuality Education is suggested to be integrated into the curriculum of Moral and Civic Education. Objectives: Help students develop positive values and attitudes towards their social and sexual relationship, including gender awareness, respecting others, protecting one’s body, getting along with the opposite sex, handling the sex impulse, and dealing with social issues relating to sex	Sexuality education is not compulsory and standardized, schools generally adopt a diverse approach, like permeating through personal and social education programs, runs once or twice a week in the form master or mistress period plus general assembly and/or extra-curricular activities. Programs are commonly atheoretical with no close link to positive youth development Evidence-based program non-existent	Forms of training: Professional development programs, and online resources Offered by: The Education Bureau; The Department of Health; and NGOs Nature: Unorganized and irregular Evaluation of training programs not commonly conducted No mandatory requirement for teacher training in sex education No systematic evaluation of teacher training	The Government and several NGOs had conducted research in investigating the effectiveness of sexuality education irregularly. The latest official survey was conducted in 2012–2013. Few rigorous evaluation studies No evaluation studies of the long-term effectiveness of sex education programs Evaluation culture not strong
**Mainland China**	2008: School-based health education policy Top-down policy without much involvement of different stakeholders	Six to seven hours Health Education is mandated in all primary, secondary and higher schools in each semester. Objectives: Discuss the issue of premarital sex; provide information on self-protection and awareness on sexual assaults, prevention and knowledge on HIV/AIDS Relatively medically-oriented	Health Education is mandated but not included in the assessment criteria, thus it is not treated seriously in some schools, and some exclude the relevant subjects in the school curriculum. Prorgams are basically atheoretical Evidence-based programs do not exist	Forms of training: Training programs Offered by: The State Education Commission (collaborated with the United Nations Population Fund); Wenhui Sex Education Correspondence Institute; Capital Normal University; National Training Center for HIV/AIDS Prevention in Schools Nature: Not systematic and nationwide; stem from the abstinence-based approach	Numerous studies on the mandatory sex education programs and training were done by scholars and different organizations, but the official evaluations conducted by the Government were insufficient. Lack of longitudinal studies on program effectiveness Evaluation culture not strong
**Taiwan**	1997: Education reform policy “The Nine-Year Joint Curriculum”	Gender education is mandated in the curriculum. Six objectives: understanding diversity in gender development understanding personal development and career planning, the possibility to overcome the limits and expectation in the society developing positive self-concept and pursuing personal interests and strengths eliminating gender discriminations and preconceptions, respecting social diversity actively seeking social support resources and establishing a gender-balanced society developing an interactive model with harmony, respect and equality between genders	Usually integrated sex education into the learning area of Health and Physical Education, Social Studies, Science and Technology, and Integrative Activities. Prorgams are basically atheoretical Evidence-based programs do not exist	Forms of training: Formal courses, talks, conferences and online resources Offered by: Government and NGOs (mainly the “Taiwan Association for Sexuality Education and the Mercy Memorial Foundation) Nature: Systematic and strictly regulated by the Government; the law requires the teachers to have corresponding qualifications in teaching the specific subject	Evaluations are organized systematically in three databases: Ministry of Science and Technology Department of Health National Digital Library of Theses and Dissertations in Taiwan Lack of longitudinal studies on program effectiveness
